# Chronic vertigo and dizziness signal unmet needs in stroke recovery

**DOI:** 10.1007/s00415-025-13562-7

**Published:** 2025-12-15

**Authors:** Lino Braadt, Markus Naumann, Dennis Freuer, Christa Meisinger, Jakob Linseisen, Michael Ertl

**Affiliations:** 1 Department of Neurology and Clinical Neurophysiology, University Hospital Augsburg, Augsburg, Germany; 2 Epidemiology, Faculty of Medicine, University of Augsburg, Augsburg, Germany; 3 Department of Neurology and Neurological Rehabilitation, District Hospital Guenzburg, Guenzburg, Germany

**Keywords:** Stroke, Vertigo, Dizziness, Complication, Rehabilitation, Recovery

## Abstract

**Introduction:**

Health-related quality of life (HRQoL) in stroke survivors often remains impaired. Chronic vertigo and dizziness are common but frequently overlooked symptoms. We investigated their impact on HRQoL and characterized symptom patterns.

**Patients and methods:**

Patients from a prospective stroke cohort study were assessed during hospitalization and followed up after three and twelve months. We evaluated functional outcomes using the modified Rankin Scale (mRS) and National Institute of Health Stroke Scale (NIHSS), and measured HRQoL with the Stroke Impact Scale (SIS). The relationship between vertigo and dizziness and the HRQoL measures was analyzed by multivariable-adjusted regression models. Subgroup analyses were performed for pre-existing and new vertigo or dizziness after stroke.

**Results:**

Of 1785 patients enrolled (mean age 69 years; 42.7% female), 988 (55%) completed the 12 months follow-up. Here, 41% reported chronic vertigo or dizziness. These symptoms significantly impacted health-related quality of life across SIS domains, with strongest effects in participation (*ß* = −11.45 (new-onset)), mobility (*ß* = −10.26 (preexisting)), and memory (*ß* = −12.80 (unspecified)), independent of age, sex, stroke severity, and comorbidities. Patients with new-onset symptoms showed higher rehabilitation participation compared to asymptomatic patients (64.3% vs 48.3%, *p* = 0.001), despite similar stroke severity (mRS: median 2 [IQR 1–3] vs 2 [1–3], *p* = 0.137; NIHSS: median 2 [IQR 0–4] vs 1 [0–4], *p* = 0.951).

**Discussion:**

Patient-reported vertigo and dizziness after acute stroke are common symptoms that impact HRQoL. Current poststroke care inadequately addresses these symptoms.

**Conclusion:**

Early identification, systematic assessment, and targeted interventions are needed.

**Supplementary Information:**

The online version contains supplementary material available at 10.1007/s00415-025-13562-7.

## Introduction

Stroke is the third-leading cause for disability-adjusted life years (DALYs) worldwide, accounting for 5.7% of total DALYs [1]. While age-standardized incidence, death, and DALYs have declined since 2000, this improvement notably slowed during the past decade (2010–19) [1]. Unfortunately, global age-standardized prevalence has risen significantly during the past decade, with projections indicating continued increases in Europe and worldwide [1, 2].

Beyond its acute manifestation and treatment, stroke must be understood as a chronic disease that fundamentally affects multiple functional systems and life dimensions. Post-stroke disability and dependency substantially reshape patients’ daily lives, impacting their social integration and independence [3–5]. Among the spectrum of post-stroke symptoms, vertigo and dizziness pose particular challenges due to their complexity and subjective nature. These symptoms contribute substantially to the disability burden in the elderly population [6, 7] and significantly impair quality of life [8, 9]. While only 3.2% of emergency room patients with vertigo/dizziness have had a stroke [10], examining the issue from the opposite perspective reveals that these symptoms affect up to 70% of stroke survivors in the chronic phase [11].

Despite the high prevalence and significant impact of vertigo and dizziness in stroke survivors, systematic diagnostic and therapeutic approaches remain insufficiently standardized in clinical practice. While vestibular rehabilitation has shown promising results in various forms [12, 13], the evidence base for therapeutic effectiveness specifically in stroke survivors remains limited [6, 12, 14, 15], as does its long-term effectiveness [16]. This gap between symptom burden and established therapeutic evidence underscores the need for more systematic investigation of post-stroke vertigo and dizziness, particularly regarding their impact on patients’ health-related quality of life.

In the present study, we investigated the impact of vertigo and dizziness in a large stroke cohort over a 12-month period. Given the high prevalence but limited evidence for specific treatment, our primary objective was to assess how these symptoms may affect patient-reported quality of life. Secondary objectives included examining associations with comorbidities and stroke risk factors, characterizing symptom frequency and patterns, and identifying the relationship between current rehabilitation participation and symptom persistence. This approach aims to enhance our understanding of post-stroke vertigo and dizziness and highlight the need for systematic assessment of these symptoms in stroke survivors.

## Methods

### Study population and data collection

**Fig. 1:**
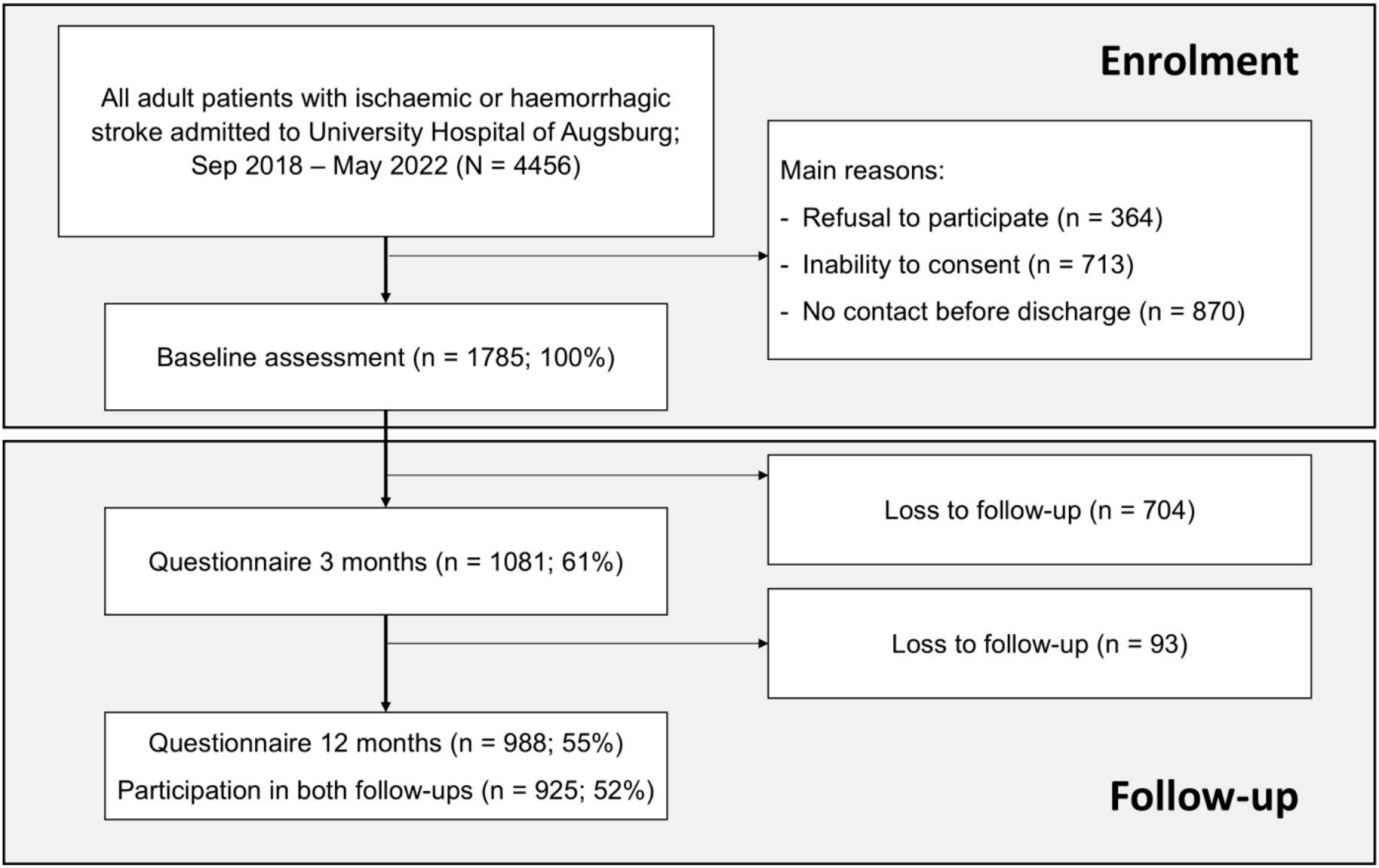
Flow chart

All adult patients admitted with ischemic or hemorrhagic strokes or transient ischemic attacks (TIA) to the University Hospital of Augsburg between September 2018 and May 2022 were screened for inclusion in the Stroke Cohort Augsburg (SCHANA) and subsequently SCHANA 2. Details of enrollment, methods, conduction of interviews as well as follow-up data have been published elsewhere for SCHANA and remained the same for SCHANA 2 [17]. In summary, study nurses recorded all stroke cases and excluded those who either refused to consent, were unable to do so, or were missed, e.g., because of premature discharge or unavailability. After having received written informed consent, 1785 patients were enrolled for baseline interviews and chart reviews. Hereby, information on sociodemographic characteristics (including age and sex, education), stroke diagnosis, laboratory findings, treatment, and comorbidities was gathered (Fig. 1).

### Exposure

Symptoms were assessed using structured interviews conducted in German during follow-up visits. Patients were asked whether they experienced dizziness (“Leiden Sie unter Schwindel?”), followed by characterization of symptom type, triggers, accompanying symptoms, duration, and frequency (days per month). The original questions are provided in Supplementary Table 1. Based on responses, we categorized symptoms as vertigo (rotational or rocking/swaying sensations; “Drehschwindel” or “Schwankschwindel”) or dizziness (gait instability or lightheadedness; “Gangunsicherheit” or “Benommenheitsgefühl”), acknowledging that the latter may have multiple potential aetiologies including non-neurological causes, such as cardiovascular or medication-related effects. Chronic symptoms were defined as those persisting at the 12-month follow-up, regardless of frequency. This definition captures patients with ongoing symptom burden, including those with episodic but recurrent symptoms (median frequency: 10 days per month for both vertigo and dizziness). Symptom frequency was captured as days per month. Analyses of vertigo or dizziness and their impacts were performed in patients who completed the second follow-up after twelve months.

### Outcome

Stroke severity was measured using the National Institutes of Health Stroke Scale (NIHSS; range 0–42) and the modified Rankin Scale (mRS; range 0–6); both were recorded upon hospital admission and discharge [18, 19]. Follow-up evaluations included the patient-reported functional outcome (range 0–4) and the quality of life after stroke, assessed with the Stroke Impact Scale (SIS), a validated stroke-specific instrument measuring eight domains: strength, hand function, activities of daily living (ADL), mobility (e.g., walking, stair climbing, transferring), communication, emotion, memory (e.g., remembering information, concentration, thinking), and participation (e.g., work, recreational activities, helping others) [20].

### Statistical analysis

Continuous variables were described by median and interquartile range (IQR) for non-normally distributed data, or by mean and standard deviation for normally distributed data. Categorical variables were reported as absolute and relative frequencies. Normal distribution was assessed using QQ-plots and the Shapiro–Wilk test. Between-group comparisons for different vertigo/dizziness groups were conducted using one-way ANOVA for normally distributed variables and Kruskal–Wallis test for non-normally distributed variables. Pairwise comparisons were performed using t-test with Welch’s correction or Mann–Whitney U tests for continuous variables and Pearson’s χ^2^-test for categorical variables. The respective effect sizes were measured using Cohen’s d, rank biserial correlation, and Cramér’s V, respectively.

To investigate the associations between health-related quality of life measures and the presence of vertigo and/or dizziness, we primarily employed multiple linear regression models. Covariates were identified using directed acyclic graphs (DAGs) from available variables to minimize confounding. Selected variables included age, sex, stroke severity (NIHSS, mRS), atrial fibrillation, and diabetes mellitus. Importantly, by adjusting for stroke severity, our analyses isolated the effect of patient-reported vertigo/dizziness on quality of life, minimizing the relevance of underlying neurological deficits. Age was modeled using natural splines with three degrees of freedom to test and account for potential non-linear relationships in all regression models. To minimize residual confounding, other continuous variables were treated as linear terms after confirming the linearity assumption. Model diagnostics included residual analysis, examination of heteroscedasticity using residual plots (predicted values vs. residuals) and the Breusch–Pagan test, assessment of multicollinearity through the variance inflation factor, evaluation of autocorrelation using the Durbin-Watson test, and identification of influential observations using the Cook’ D statistic. As some deviations from model assumptions were observed, we conducted two complementary regression analyses to evaluate the robustness of our findings: robust regression using MM-estimation with HC1 standard errors and median regression (quantile regression at *τ* = 0.5). The consistency of results across these three approaches was assessed. For vertigo/dizziness comparisons, *p* values were adjusted for multiple testing using the Benjamini–Hochberg false discovery rate (FDR) method within each regression approach. The Benjamini–Hochberg method was chosen over Bonferroni correction to balance Type I error control with statistical power, particularly important given the exploratory nature of domain-specific quality of life assessments and the clinical relevance of potentially missed effects in this patient population. To examine potential bias from missing rehabilitation participation data (9%, *n* = 89), we compared respondents and non-respondents on characteristics like age, stroke severity on discharge (NIHSS, mRS), sex, and educational background. Finding no significant differences, we concluded that these data were Missing Completely At Random (MCAR) and proceeded with complete case analysis. Separately, for vertigo and dizziness variables assessed at twelve months post-stroke, the proportion of missing values was low (3.3%, *n* = 33), allowing us to similarly employ complete case analyses for these outcomes.

Statistical tests were performed two-sided at the significance threshold *α* = 0.05. Statistical analyses and plots were performed with SPSS (version 29.0.0.0) and R (version 4.4.2), respectively.

The STROBE cohort reporting guidelines were used in the present study [21].

## Results

### Study population and follow-up

Of the initial cohort, 988 patients completed the twelve-month follow-up, while 797 (45%) did not participate. Those completing follow-up had experienced less severe strokes, as evidenced by NIHSS and mRS during both initial assessment and at the time of discharge. Demographics were similar between these two groups, see Supplementary Table 2.

### Temporal patterns

At the 12-month follow-up, 403 (40.8%) of 988 participating patients reported vertigo or dizziness. Sensitivity analyses indicated a potential population prevalence of 22.6–65.8%, accounting for dropout scenarios.

Age, sex distribution, and rehabilitation participation differed significantly across symptom duration groups (see Table 1). Patients with preexisting symptoms were notably older than those with post-stroke onset or no symptoms. Most importantly, functional outcomes at 12 months varied substantially (*p* = 2.0 × 10^−22^): patients without vertigo or dizziness showed better functional outcomes than all symptomatic groups (post-stroke: *p*_adj_ = 3.5 × 10^−17^; preexisting: *p*_adj_ = 2.5 × 10^−10^; unspecified: *p*_adj_ = 5.8 × 10^−7^). Among symptomatic patients, those with post-stroke symptoms reported worse functional outcomes compared to those with preexisting symptoms (*p*_adj_ = 0.023). Notably, this poorer functional outcome occurred despite a higher rehabilitation participation rate of those with post-stroke symptoms compared to those without such symptoms (64.3% vs. 48.3%, *p* = 0.001, OR 1.93).

**Table 1:** Comparison of patients reporting vertigo, dizziness, or no symptoms 12 months after stroke (*N* = 950)

Characteristic	Measure	Total	No vertigo/dizziness	New vertigo/dizziness	Preexisting vertigo/dizziness	Unspecified vertigo/dizziness	Statistical test
Age (years)	*n* (valid)	950	549	136	171	94	
	*M* (*SD*)	68.4 (12.3)	66.8 (12.2)	68.6 (11.8)	71.8 (11.9)	70.9 (12.8)	***p* = 8.3 × 10**^**−**^**⁶**^**b**^
Sex	Male	568 (59.5%)	355 (64.3%)	78 (57.4%)	94 (55.0%)	41 (42.7%)	***p* = 3.9 × 10**^**−4a**^
	Female	387 (40.5%)	197 (35.7%)	58 (42.6%)	77 (45.0%)	55 (57.3%)	
Rehabilitation	No	420 (48.1%)	259 (51.7%)	45 (35.7%)	76 (47.2%)	40 (47.1%)	***p* = 0.015**^a^
	Yes	453 (51.9%)	242 (48.3%)	81 (64.3%)	85 (52.8%)	45 (52.9%)	
NIHSS at admission	*n* (valid)	903	524	125	160	94	
	*Mdn* (Q1-Q3)	2.0 (0.0–4.0)	1.0 (0.0–4.0)	2.0 (0.0–4.0)	2.0 (0.0–4.0)	2.0 (1.0–5.8)	*p* = 0.056^c^
NIHSS at discharge	*n* (valid)	855	497	120	155	83	
	*Mdn* (Q1-Q3)	0.0 (0.0–2.0)	0.0 (0.0–2.0)	0.0 (0.0–2.0)	0.0 (0.0–2.0)	0.0 (0.0–1.5)	*p* = 0.978^c^
mRS at admission	*n* (valid)	903	525	124	160	94	
	*Mdn* (Q1-Q3)	2.0 (1.0–3.0)	2.0 (1.0–3.0)	2.0 (1.0–3.0)	2.0 (1.0–3.0)	3.0 (1.0–4.0)	***p* = 0.005**^c^
mRS at discharge	*n* (valid)	900	521	126	160	93	
	*Mdn* (Q1-Q3)	1.0 (0.0–2.0)	1.0 (0.0–2.0)	1.0 (0.0–2.0)	1.0 (0.0–2.0)	1.0 (0.0–2.0)	***p* = 0.050**^c^
Functional outcome after 12 months	*n* (valid)	913	532	127	166	88	
	*Mdn* (Q1-Q3)	1.0 (0.0–2.0)	1.0 (0.0–2.0)	2.0 (1.0–3.0)	2.0 (1.0–3.0)	2.0 (1.0–3.0)	***p* = 2.0 × 10**^**−22c**^
Stroke etiology	Macroangiopathy	184 (20.4%)	104 (20.1%)	33 (25.6%)	31 (19.0%)	16 (17.8%)	*p* = 0.236^a^
	Cardiogenic	223 (24.8%)	117 (22.6%)	29 (22.5%)	47 (28.8%)	30 (33.3%)	
	Microangiopathy	166 (18.4%)	96 (18.5%)	30 (23.3%)	29 (17.8%)	11 (12.2%)	
	Other etiology	22 (2.4%)	14 (2.7%)	2 (1.6%)	5 (3.1%)	1 (1.1%)	
	Cryptogenic	305 (33.9%)	187 (36.1%)	35 (27.1%)	51 (31.3%)	32 (35.6%)	

Groups compared in statistical tests: No vertigo/dizziness vs. New vertigo/dizziness vs. Preexisting vertigo/dizziness vs. Unspecified Vertigo/dizziness with significant *p* values in bold

*ANOVA* Analysis of Variance, *M* mean, *Mdn* median, *mRS* modified Rankin Scale, *NIHSS* National Institutes of Health Stroke Scale, *Q* quartile, *SD* standard deviation

^a^Statistical tests: Chi-square test

^b^ANOVA

^c^Kruskal-Wallis test

Initial stroke severity, measured by NIHSS at admission, showed borderline significant differences (*p* = 0.056), with patients reporting unspecified symptom duration showing worse severity compared to other groups. No significant differences were observed in NIHSS at discharge (*p* = 0.978) and stroke etiology (*p* = 0.2364).

### Rehabilitation participants

While patients who participated in rehabilitation programs did not differ significantly in age or sex, they did in terms of stroke severity. Participants suffered more severe strokes compared to those who did not participate in such programs, both at admission (NIHSS *p*_adj_ = 1.3 × 10^−25^, mRS *p*_adj_ = 2.9 × 10^−26^) and at discharge (NIHSS *p*_adj_ = 1.2 × 10^−36^, mRS *p*_adj_ = 3.5 × 10^−46^), see Supplementary Table 3.

Patients with post-stroke symptoms and preexisting symptoms did not differ in their symptom characteristics, with dizziness being reported equally often (44.3% post-stroke vs. 55.7% preexisting, *p* = 0.747, OR 0.916), as they did report equally many symptomatic days per month (median 10 [IQR 5–18.5] vs. 10 [IQR 5–25], *p* = 0.302).

### Characteristics of symptoms

Among patients at 12 months follow-up, 288 (29.1%) reported dizziness and 97 (9.8%) reported vertigo. Detailed comparisons between these groups are presented in Table 2. Patients with dizziness were significantly older and reported more symptomatic days per month compared to those with vertigo. While stroke severity at admission showed borderline differences, patients with dizziness had significantly worse functional outcomes at 12 months.

**Table 2:** Comparison of patients by vertigo and dizziness symptom status 12 months after stroke (*N* = 950)

Characteristic	Measure	Total	Isolated vertigo	Dizziness	Statistical test
Age (years)	*n* (valid)	383	96	287	
	*M* (*SD*)	70.5 (12.1)	66.0 (13.9)	72.1 (11.1)	***p* = 1.4 × 10**^−**4a**^
Sex	male	205 (53.2%)	43 (44.3%)	162 (56.2%)	*p* = 0.055^c^
	female	180 (46.8%)	54 (55.7%)	126 (43.8%)	
NIHSS at admission	*n* (valid)	363	94	269	
	*Mdn* (Q1-Q3)	2.0 (0.0–4.0)	1.0 (0.0–3.0)	2.0 (1.0–4.0)	*p* = 0.061^b^
NIHSS at discharge	*n* (valid)	341	91	250	
	*Mdn* (Q1-Q3)	0.0 (0.0–2.0)	0.0 (0.0–1.0)	0.0 (0.0–2.0)	*p* = 0.250^b^
mRS at admission	*n* (valid)	360	93	267	
	*Mdn* (Q1-Q3)	2.0 (1.0–4.0)	2.0 (1.0–3.0)	3.0 (1.0–4.0)	*p* = 0.057^b^
mRS at discharge	*n* (valid)	361	94	267	
	*Mdn* (Q1-Q3)	1.0 (0.0–2.0)	1.0 (0.0–2.0)	1.0 (0.0–2.0)	*p* = 0.058^b^
Functional outcome after 12 months	*n* (valid)	365	90	275	
	*Mdn* (Q1-Q3)	2.0 (1.0–3.0)	1.5 (0.0–2.8)	2.0 (1.0–3.0)	***p* = 0.010**^**b**^
Symptomatic days per month	*n* (valid)	385	97	288	
	*Mdn* (Q1-Q3)	10.0 (4.0–20.0)	8.0 (3.0–15.0)	10.0 (5.0–20.0)	***p* = 0.010**^**b**^

Groups compared in statistical tests: Isolated vertigo vs. Dizziness reported 12 months after stroke with significant *p* values in bold

*M* mean, *Mdn* median, *mRS* modified Rankin Scale, *NIHSS* National Institutes of Health Stroke Scale, *Q* quartile, *SD* standard deviation

^a^Statistical tests: *t*-test

^b^Mann-Whitney test

^c^Chi-square test

### Quality of life

**Fig. 2:**
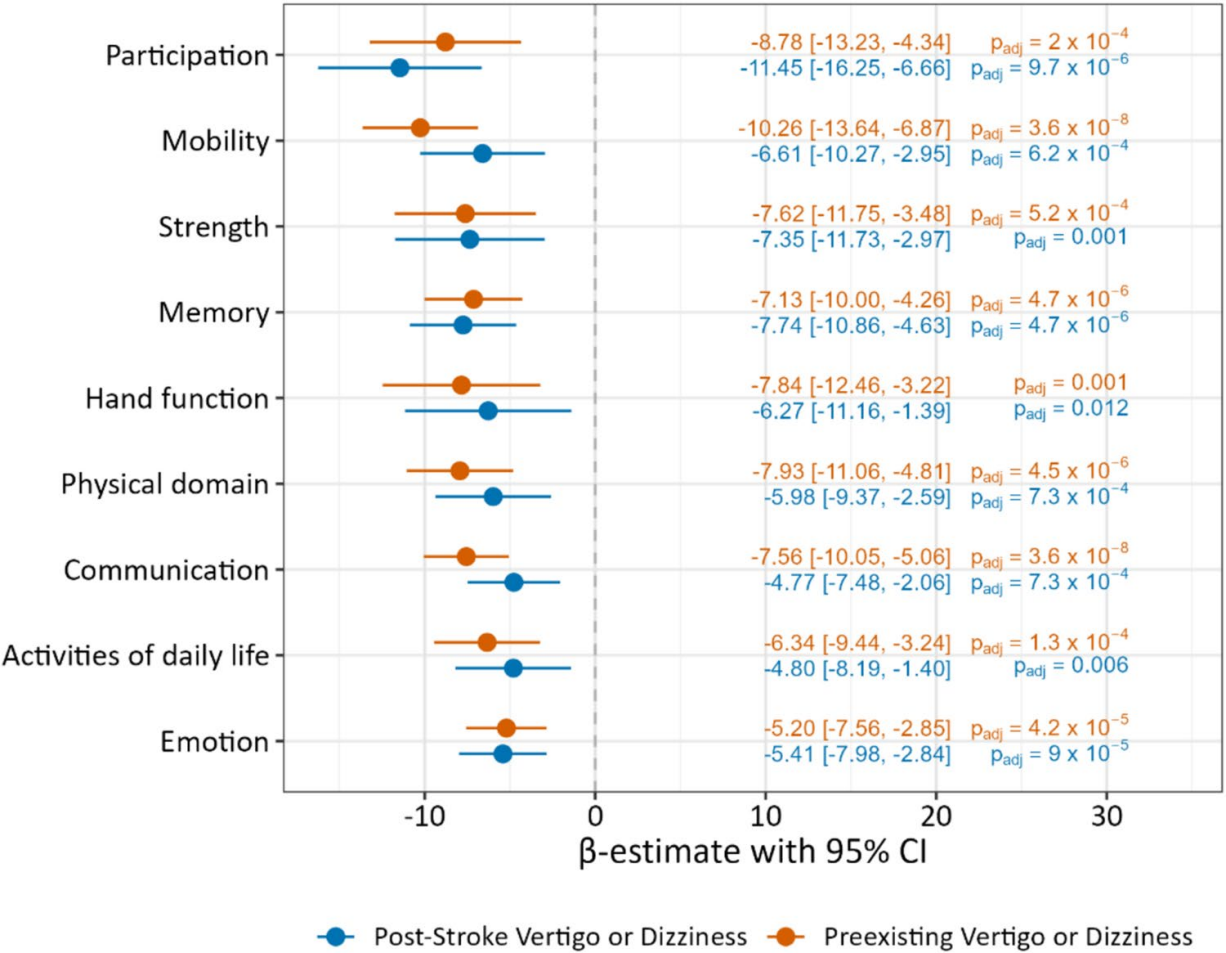
Health-related quality of life in patients with post-stroke versus preexisting vertigo or dizziness 12 months after stroke

Health-related quality of life, as measured by SIS domains, showed distinct patterns across different symptom groups (Fig. 2). Multiple linear regression analyses, adjusting for sex, age, stroke severity, diabetes mellitus, and atrial fibrillation, revealed domain-specific associations:

The participation domain ($R_{{{\mathrm{adj}}}}^{2}$ = 0.15) showed the strongest impairment in patients with post-stroke symptoms (*β* = −11.45, 95% CI [−16.25; −6.66], *p*_adj_ = 9.7 × 10^−6^). While patients with preexisting vertigo or dizziness also showed substantial impairment in this domain (*β* = −8.78, 95% CI [−13.23; −4.34], *p*_adj_ = 2 × 10^−4^), their most severe impact was observed in the domain mobility ($R_{{{\mathrm{adj}}}}^{2}$ = 0.25; *β* = −10.26, 95% CI [−13.64; −6.87], *p*_adj_ = 3.6 × 10^−8^). The post-stroke group showed less severe impairment in the mobility domain (*β* = −6.61 [−10.27; −2.95], *p*_adj_ = 6.2 × 10^−4^). Patients who did not specify the duration of their symptoms showed the worst health-related quality of life in the SIS-domain memory (*β* = −12.80 [−16.42; −9.19], *p*_adj_ = 6.4 × 10^−11^; $R_{{{\mathrm{adj}}}}^{2}$ = 0.14), while patients with post-stroke onset of symptoms (*β* = −7.74 [−10.86; −4.63], *p*_adj_ = 4.7 × 10^−6^) and those with preexisting symptoms (*β* = −7.13 [−10.00; −4.26], *p*_adj_ = 4.7 × 10^−6^) showed less severe outcomes in this domain (see Supplementary Fig. 1). For comparison of the linear regression model with sensitivity analyses, see Supplementary Fig. 2.

## Discussion

This study provides evidence that patient-reported vertigo and dizziness at twelve months following stroke significantly affect patients’ health-related quality of life, with comparable effects observed in patients with preexisting symptoms. All symptom groups showed reduced scores across all SIS domains with distinct patterns of domain-specific impairment. Notably, patients with post-stroke vertigo or dizziness showed poorer quality of life outcomes despite higher rehabilitation participation and similar initial stroke severity compared to those without such symptoms.

While chronic vertigo and dizziness are known to impair quality of life [8, 9], the Stroke Impact Scale provided novel insights into domain-specific impairments in stroke survivors, revealing distinct patterns between post-stroke and preexisting symptoms. Patients with post-stroke symptoms showed particular impairment in the participation domain. This aligns with previous findings in vestibular disorders where activity-specific impairments and reduced self-efficacy led to avoidance behaviors, potential loss of workforce and isolation [22, 23], with direct implications for occupational reintegration and social functioning. In contrast, patients with preexisting symptoms showed their worst outcomes in the SIS-domain mobility, potentially reflecting existing comorbidities and multisensory deficits in this older patient group [24].

The paradoxical relationship between rehabilitation participation and outcomes provides important insight into the clinical challenges of managing post-stroke vertigo and dizziness. Our findings suggest that current therapeutic strategies may not fully address the complex needs of these patients, indicating that further individualization might be necessary in rehabilitation programs [25]. This aligns with evidence that current rehabilitation approaches for community participation enhancement often fail to capture all relevant aspects of these complex needs or show limitations in their interpretability [26].

The etiology of vertigo and dizziness after stroke is complex and multifactorial. Beyond infratentorial lesions in the posterior circulation affecting vestibular projections, symptoms may originate from a broader processing network including right hemispheric cortical regions [27, 28], termed central vertigo network [29]. Less specifically, bihemispheric small vessel disease [30], peripheral nervous system disorders, cardiovascular conditions (including cardiac arrhythmias and blood pressure dysregulation), psychiatric disease, or visual impairments [31], can contribute to and exacerbate these symptoms [32]. This complexity, reflected in the variable relationship between vestibular function tests and patient-reported symptom intensity [33], emphasizes the importance of a thorough assessment considering multiple possible aetiologies [34].

Given the significant impact of vertigo and dizziness twelve months after stroke on health-related quality of life and their multifactorial nature, a systematic approach to post-stroke care is essential. This should include careful history-taking, neurological examination, and consideration of cardiovascular as well as psychiatric comorbidities [35–41]. Notably, patients with dizziness were older and predominantly male compared to those with vertigo, highlighting the importance of individualized assessment approaches.

The high prevalence of vertigo and dizziness twelve months after stroke (40.8%) reinforces the need for systematic screening during follow-up visits. Neurologists and rehabilitation specialists should remain vigilant for these symptoms, which may otherwise be unaddressed. The domain-specific impairments suggest that rehabilitation approaches benefit from further individualization: older patients with preexisting vertigo or dizziness might benefit from mobility-focused interventions, while patients with new-onset symptoms require particular attention to reintegration and participation.

## Strengths and limitations

The prospective cohort study design with twelve-month follow-up and detailed assessment of stroke-specific quality of life is a major strength of this study, providing insights into clinically relevant outcomes.

Some limitations warrant consideration. First, only 988 out of 1785 patients (55%) completed twelve-month follow-up, presenting a potential attrition bias. Patients completing follow-up had less severe strokes, potentially underestimating the true impact in more severely affected patients. Second, specific details about rehabilitation types and treatments were not captured. Third, the retrospective nature of symptom onset reporting introduces a potential recall bias and our symptom assessment relied on patient reports without standardized vestibular assessment tools or objective testing, limiting differentiation of central from peripheral causes and objective symptom quantification. Fourth, we lack information on stroke lesion localization, particularly posterior circulation involvement, which is known to be possibly associated with vertigo. Finally, our single center cohort in Germany may limit generalizability.

Future research should include systematic screening with prospective intervention studies, comprehensive multidisciplinary assessment, and individualized rehabilitation approaches.

## Conclusion

Vertigo and dizziness at 12 months after stroke substantially impair health-related quality of life with domain-specific patterns: participation for post-stroke onset, mobility for those with preexisting symptoms. The paradoxical finding of poorer outcomes despite higher rehabilitation participation reveals gaps in current post-stroke care. Systematic screening, comprehensive multidisciplinary assessment, and individualized rehabilitation are needed to address this often-overlooked symptom burden.

## Supplementary Information

Below is the link to the electronic supplementary material.

Supplementary file1 (DOCX 468 KB)

Supplementary file2 (DOCX 31 KB)

## Data Availability

The datasets used and analyzed during the current study are available from the corresponding author on reasonable request.
