# Supplementary material for: Chronic vertigo and dizziness signal unmet needs in stroke recovery
Source: J Neurol. 2025 Dec 15;273(1):26. doi: 10.1007/s00415-025-13562-7 (PMC12705797; doi:10.1007/s00415-025-13562-7)
Supplement: Supplementary file 1 — Supplementary file1 (DOCX 468 KB) [file 415_2025_13562_MOESM1_ESM.docx]

**Chronic Vertigo and Dizziness Signal Unmet Needs in Stroke Recovery**

Lino Braadt^1^, Markus Naumann^1^, Dennis Freuer^2^, Christa Meisinger^2^, Jakob Linseisen^2^, Michael Ertl^1,3^

^1^Department of Neurology and Clinical Neurophysiology, University Hospital Augsburg, Augsburg, Germany, ^2^Epidemiology, Faculty of Medicine, University of Augsburg, Augsburg, Germany, ^3^Department of Neurology and Neurological Rehabilitation, District Hospital Guenzburg, Guenzburg, Germany

**Correspondence:** Lino Braadt, [lino-dominic.braadt@uk-augsburg.de](mailto:lino-dominic.braadt@uk-augsburg.de); Tel.: +49 821/400-2991

**Supplementary Material**

This supplementary material contains two additional figures:

Supplementary Figure 1 presents health-related quality of life outcomes in patients with vertigo or dizziness of unspecified duration.

Supplementary Figure 2 shows sensitivity analysis comparing linear, median, and robust regression models for all SIS domain scores across the three patient groups.


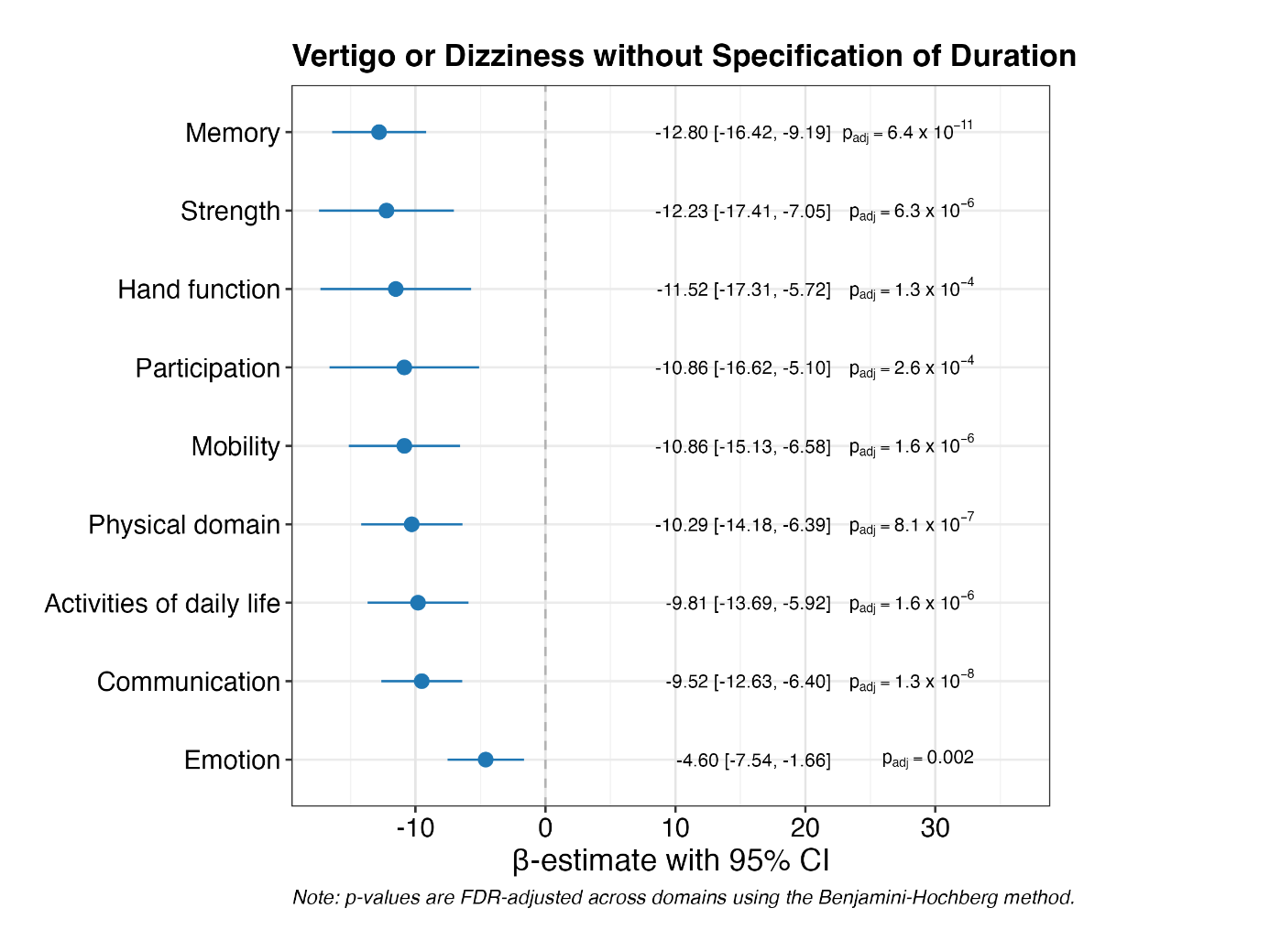


Supplementary Figure 1 | Health-related Quality of Life in Patients with Vertigo or Dizziness, without Specification of Duration


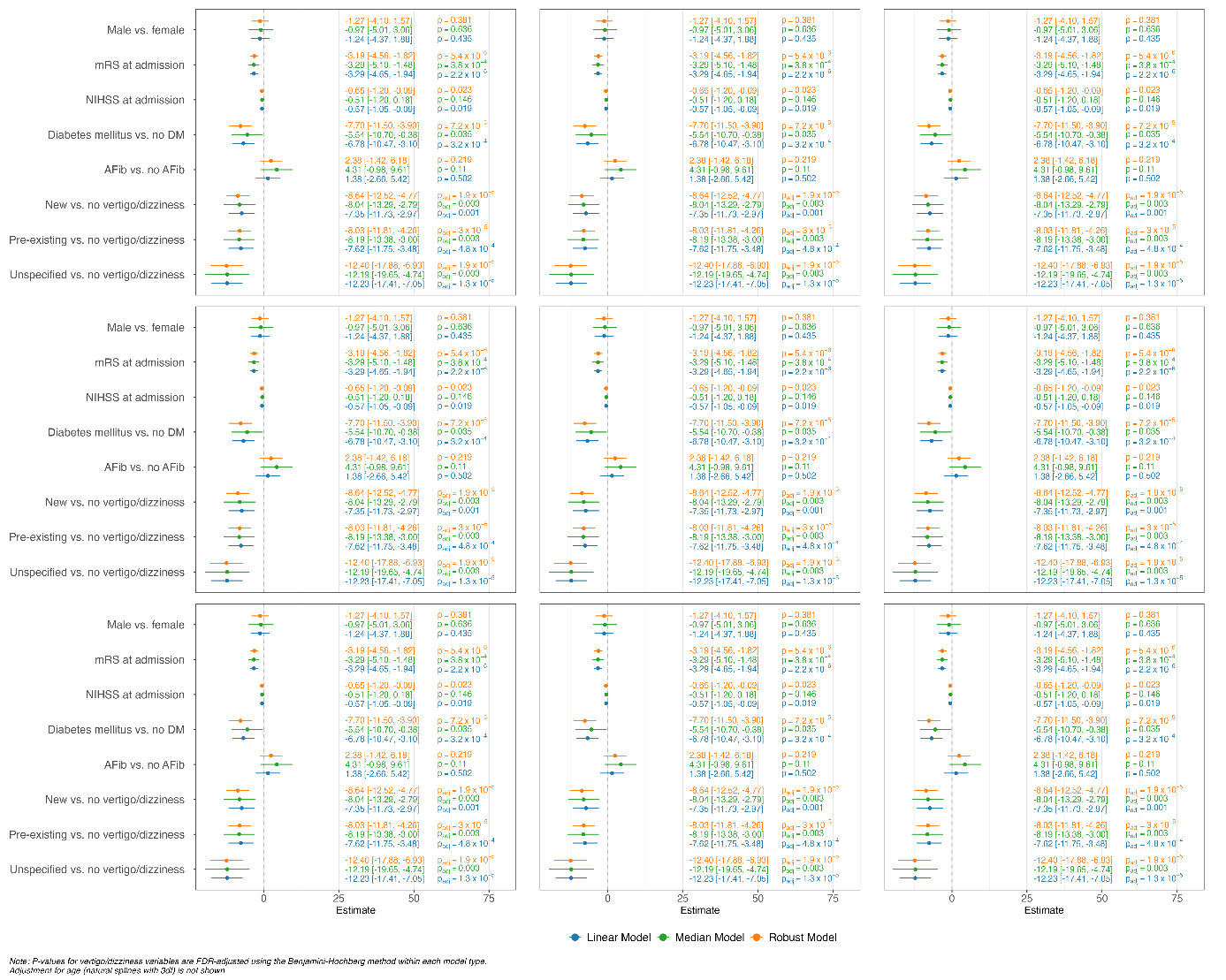


Supplementary Figure 2 | Comparison of Regression Models for Health-Related Quality of Life Outcomes in Patients with Vertigo and Dizziness
