# Supplementary material for: Chronic vertigo and dizziness signal unmet needs in stroke recovery
Source: J Neurol. 2025 Dec 15;273(1):26. doi: 10.1007/s00415-025-13562-7 (PMC12705797; doi:10.1007/s00415-025-13562-7)
Supplement: Supplementary file 2 — Supplementary file2 (DOCX 31 KB) [file 415_2025_13562_MOESM2_ESM.docx]

**Chronic Vertigo and Dizziness Signal Unmet Needs in Stroke Recovery**

Lino Braadt^1^, Markus Naumann^1^, Dennis Freuer^2^, Christa Meisinger^2^, Jakob Linseisen^2^, Michael Ertl^1,3^

^1^Department of Neurology and Clinical Neurophysiology, University Hospital Augsburg, Augsburg, Germany, ^2^Epidemiology, Faculty of Medicine, University of Augsburg, Augsburg, Germany, ^3^Department of Neurology and Neurological Rehabilitation, District Hospital Guenzburg, Guenzburg, Germany

**Correspondence:** Lino Braadt, [lino-dominic.braadt@uk-augsburg.de](mailto:lino-dominic.braadt@uk-augsburg.de); Tel.: +49 821/400-2991

**Supplementary Material**

This supplementary material contains three additional tables:

Supplementary Table 1 | Vertigo and Dizziness Questionnaire

| Leiden Sie unter Schwindel? | Ja  Nein |
| --- | --- |
| Welche Art von “Schwindel” haben Sie? | Schwankschwindel („wie auf dem Schiff“)  Drehschwindel („wie Karussell fahren“)  Gangunsicherheit  Benommenheitsgefühl  Andere |
| Gibt es Auslöser? | Kopfbewegung  Aufsetzen/Aufstehen  Beim Gehen  Visuelle Reize  Stress  Bestimmte Umgebungssituationen (z.B. Einkaufen, enge Räume)  Husten, Pressen, Niesen  Andere |
| Gibt es begleitende Beschwerden? | Übelkeit/Erbrechen  Sehstörungen/Doppelbilder  Stand-/Gangunsicherheiten  Sturzneigung  Kopfschmerzen  Hörminderung  Tinnitus/Ohrgeräusche  Licht- oder Lärmempfindlichkeit  Herzrasen  Atemnot  Schwitzen  Angst  Andere |
| Wie lange leiden Sie an den Schwindelbeschwerden? | Geben Sie bitte die entsprechende Anzahl in Monaten / Jahren an: |
| An wie vielen Tagen pro Monat leiden Sie durchschnittlich an den Schwindelbeschwerden? | Geben Sie bitte die Anzahl der Tage pro Monat an: |
| Wie lange halten die Schwindelbeschwerden an? | Sekunden  Minuten  Stunden  Tage |

Supplementary Table 2 | Comparison of Follow-up Participants

| Characteristic | Measure | Total | Follow-ups not completed | Follow-up 2 completed | Statistical Test |
| --- | --- | --- | --- | --- | --- |
| Age (years) | *n* (valid) | 1755 | 772 | 983 |  |
|  | *M* (*SD*) | 69.1 (13.1) | 69.8 (14.2) | 68.6 (12.3) | *p* = 0.072 ^a^ |
| Sex | male | 1014 (57.3%) | 429 (54.9%) | 585 (59.2%) | *p* = 0.079 ^c^ |
|  | female | 755 (42.7%) | 352 (45.1%) | 403 (40.8%) |  |
| NIHSS at admission | *n* (valid) | 1676 | 738 | 938 |  |
|  | *Mdn* (Q1-Q3) | 2.0 (0.0-4.0) | 2.0 (0.0-5.0) | 2.0 (0.0-4.0) | ***p* = 0.012 ^b^** |
| NIHSS at discharge | *n* (valid) | 1590 | 705 | 885 |  |
|  | *Mdn* (Q1-Q3) | 0.0 (0.0-2.0) | 1.0 (0.0-2.0) | 0.0 (0.0-2.0) | ***p* = 7.4×10⁻^5^ ^b^** |
| mRS at admission | *n* (valid) | 1675 | 740 | 935 |  |
|  | *Mdn* (Q1-Q3) | 2.0 (1.0-3.0) | 2.0 (1.0-4.0) | 2.0 (1.0-3.0) | ***p* = 0.003 ^b^** |
| mRS at discharge | *n* (valid) | 1676 | 745 | 931 |  |
|  | *Mdn* (Q1-Q3) | 1.0 (0.0-2.0) | 1.0 (0.0-3.0) | 1.0 (0.0-2.0) | ***p* = 3.8×10⁻⁷ ^b^** |
| *Statistical tests: t-test* ^a^*, Mann-Whitney test* **^b^***, Chi-square test* ^c^ | | | | | |
| *Groups compared in statistical tests: Follow-ups not completed vs. Follow-up 2 completed*  *Abbreviations: M – mean, Mdn – median, mRS – modified Rankin Scale, n – number, NIHSS – National Institutes of Health Stroke Scale, Q – quartile, SD – standard deviation* | | | | | |

Supplementary Table 3 | Comparison of patients according to rehabilitation participation

| Characteristic | Measure | Total | No Rehabilitation | Rehabilitation | Statistical Test |
| --- | --- | --- | --- | --- | --- |
| Age (years) | *n* (valid) | 894 | 430 | 464 |  |
|  | *M* (*SD*) | 68.6 (12.3) | 69.1 (12.2) | 68.1 (12.3) | *p* = 0.244 ^a^ |
| Sex | male | 535 (59.5%) | 267 (61.9%) | 268 (57.3%) | *p* = 0.173 ^c^ |
|  | female | 364 (40.5%) | 164 (38.1%) | 200 (42.7%) |  |
| NIHSS at admission | *n* (valid) | 853 | 409 | 444 |  |
|  | *Mdn* (Q1-Q3) | 2.0 (0.0-4.0) | 1.0 (0.0-2.0) | 3.0 (1.0-5.0) | ***p* = 1.3×10⁻²⁵ ^b^** |
| NIHSS at discharge | *n* (valid) | 800 | 396 | 404 |  |
|  | *Mdn* (Q1-Q3) | 0.0 (0.0-2.0) | 0.0 (0.0-0.0) | 1.0 (0.0-3.0) | ***p* = 1.2×10⁻³⁶ ^b^** |
| mRS at admission | *n* (valid) | 850 | 406 | 444 |  |
|  | *Mdn* (Q1-Q3) | 2.0 (1.0-3.0) | 1.0 (0.0-3.0) | 3.0 (2.0-4.0) | ***p* = 2.9×10⁻²⁶ ^b^** |
| mRS at discharge | *n* (valid) | 844 | 405 | 439 |  |
|  | *Mdn* (Q1-Q3) | 1.0 (0.0-2.0) | 0.0 (0.0-1.0) | 2.0 (1.0-3.0) | ***p* = 3.5×10⁻⁴⁶ ^b^** |
| Functional outcome after 12 months | *n* (valid) | 861 | 421 | 440 |  |
|  | *Mdn* (Q1-Q3) | 1.0 (0.0-2.0) | 0.0 (0.0-1.0) | 2.0 (1.0-3.0) | ***p* = 4.5×10⁻³⁵ ^b^** |
| Stroke aetiology | Macroangiopathy | 174 (20.5%) | 77 (19.0%) | 97 (21.9%) | ***p* = 0.008 ^c^** |
|  | Cardiogenic | 204 (24.1%) | 100 (24.7%) | 104 (23.5%) |  |
|  | Microangiopathy | 156 (18.4%) | 58 (14.3%) | 98 (22.1%) |  |
|  | Other aetiology | 22 (2.6%) | 10 (2.5%) | 12 (2.7%) |  |
|  | Cryptogenic | 292 (34.4%) | 160 (39.5%) | 132 (29.8%) |  |
| *Statistical tests: t-test* ^a^*, Mann-Whitney test* ^b^*, Chi-square test* ^c^  *Groups compared in statistical tests: No Rehabilitation vs. Rehabilitation*  *Abbreviations: M – mean, Mdn – median, mRS – modified Rankin Scale, n – number, NIHSS – National Institutes of Health Stroke Scale, Q – quartile, SD – standard deviation* | | | | | |
